# Temporal changes in ART initiation in adults with high CD4 counts in Latin America: a cohort study

**DOI:** 10.1002/jia2.25413

**Published:** 2019-12-19

**Authors:** Brenda E Crabtree‐Ramírez, Yanink Caro‐Vega, Pablo F Belaunzarán‐Zamudio, Bryan E Shepherd, Peter F Rebeiro, Valdilea Veloso, Claudia P Cortes, Denis Padgett, Eduardo Gotuzzo, Juan Sierra‐Madero, Catherine C McGowan, Anna K Person, on behalf of the Caribbean, Central, South America Network for HIV Epidemiology (CCASAnet)

**Affiliations:** ^1^ Departamento de Infectología Instituto Nacional de Ciencias Médicas y Nutrición México City Mexico; ^2^ Vanderbilt University School of Medicine Nashville TN USA; ^3^ Instituto de Pesquisa Clínica Evandro Chagas Fundacão Oswaldo Cruz Rio de Janeiro Brazil; ^4^ Facultad de Medicina de la Universidad de Chile and Fundación Arriarán Santiago de Chile Chile; ^5^ Instituto Hondureño de Seguro Social and Hospital Escuela Universitario Tegucigalpa Honduras; ^6^ Instituto de Medicina Tropical Alexander von Humboldt Lima Peru

**Keywords:** ARV, cohort studies, HIV care continuum, Latin America and the Carribean, Adherence, linkage to care

## Abstract

**Introduction:**

In 2013, the World Health Organization (WHO) recommended initiating combination ART (cART) in all adults with HIV and CD4+ lymphocyte counts (CD4) <500 cells/mm^3^. In 2015, this was updated to recommend cART initiation in all patients with HIV, regardless of CD4 count. Implementation of these guidelines in real‐world settings has not been evaluated in Latin America. To assess changes in time to cART initiation during routine care, we estimated trends in time from enrolment in care to cART initiation in HIV‐positive adults with high CD4 counts in the Caribbean, Central and South America network for HIV Epidemiology (CCASAnet) during 2003 to 2017.

**Methods:**

All cART‐naive individuals ≥18 years of age from 2003 to 2017 with CD4 ≥350 cells/mm^3^ and without AIDS at enrolment at five CCASAnet sites (Brazil, Chile, Honduras, Mexico and Peru) were included. Patients without information regarding AIDS‐defining events were excluded. We estimated unadjusted median time from enrolment to cART initiation by calendar year using Kaplan‐Meier methods and calculated adjusted hazard ratios (HR) and 95% confidence intervals (95% CI) for trends in cART initiation using Cox models and restricted cubic splines for continuous variables, accounting for age, sex, CD4 at enrolment, route of HIV transmission and clinic site.

**Results:**

Of the 3171 patients included, 1,650 (52%) had CD4 ≥500 cells/mm^3^ at enrolment. Median time to cART initiation after 2013 was 6.21 weeks (interquartile range (IQR): 1.89, 23.21), and 4.71 weeks (IQR: 1.43, 9.57) after 2015. Among 763 (24%) patients who never initiated cART, 33 (4.3%) were reported as deceased, 481 (63%) were lost to follow‐up, and 249 (33%) were administratively censored before initiation. Adjusted probability of cART initiation greatly increased in recent years, in particular after 2013 and 2015 (2013 vs. 2003: HR = 7.14; 95% CI: 5.84 to 8.73, and 2015 vs. 2003: HR = 12.60; 95% CI: 10.37 to 15.32).

**Conclusions:**

Time to cART initiation decreased substantially, roughly following changes in WHO guidelines in this real‐world setting in Latin America. However, a very high proportion of patients never started cART, compromising retention in care and survival, as shown by their higher proportion of LTFU and death, which reinforce the notion that earlier treatment implementation strategies are needed.

## 1 Introduction

Combined antiretroviral therapy (cART) has dramatically improved the survival of people living with HIV/AIDS (PLWHA) and in more recent years, prompt cART initiation after HIV diagnosis has been demonstrated to favourably influence clinical, virologic, immunologic and retention‐in‐care outcomes. Due to the undesirable side effects and toxicities of many older antiretroviral medications, cART initiation early in the epidemic was generally deferred among asymptomatic patients with CD4 counts >200 cells/mm^3^ 1. In 2013, thanks to mounting evidence in observational studies and randomized controlled trials 2, 3, 4, the World Health Organization (WHO) recommended expanding the eligibility criteria for cART initiation to those with CD4 counts ≤500 cells/mm^3^, with special priority given to individuals with severe or advanced HIV disease (WHO clinical stages 3 or 4) and those with CD4 counts ≤350 cells/mm^3^ 5. Later, two randomized clinical trials (START and TEMPRANO) confirmed the beneficial effects of immediate cART initiation regardless of CD4 counts in a broad population of HIV‐positive patients 6, 7, providing further evidence for the revised 2015 WHO guidelines recommending universal ART initiation 8. More recently, several randomized clinical trials (RapIT, RAPID and a trial conducted by the GHESKIO group in Haiti) 9, 10, 11 have also shown the benefits of immediate or same‐day ART initiation in asymptomatic patients, in turn prompting the WHO to issue rapid cART initiation guidelines in 2017 12.

Although available evidence in Latin America buttresses local guidelines recommending universal cART initiation, its implementation remains uneven throughout the region 13. Even though reports show trends towards lower rates of late cART initiation in recent years, the prevalence of late cART initiation (<200 cell/mm^3^ counts and/or AIDS‐defining illness at initiation) is still high, mainly due to barriers for broader and universal HIV testing and cART initiation 13. For example, in Mexico it is estimated that only 60% of HIV‐positive patients are diagnosed 14. Indeed, high rates of late cART initiation have been reported across Latin America 13, 15, 16, 17. Delays in cART initiation among asymptomatic patients may reflect barriers to prompt cART initiation in the healthcare system that are worth addressing. As such, the aim of this study was to assess temporal changes in cART initiation after enrolment in routine HIV care in Latin America.

## 2 Methods

CCASAnet (Caribbean, Central and South America network for HIV Epidemiology, http://ccasanet.vanderbilt.edu) has been described elsewhere 16, 18. The collaboration was established in 2006 as Region 2 of IeDEA (International epidemiology Databases to Evaluate AIDS, https://www.iedea.org/) with the purpose of collecting HIV data from Central and South America and the Caribbean to describe unique characteristics of the HIV epidemic in this region. Five CCASAnet sites contributed data to this study: Instituto Nacional de Infectología Evandro Chagas, Fundacão Oswaldo Cruz, Rio de Janeiro, Brazil (Fiocruz‐Brazil); Fundación Arriarán, Santiago, Chile (FA‐Chile); Instituto Hondureño de Seguridad Social and Hospital Escuela Universitario, Tegucigalpa, Honduras (IHSS/HE‐Honduras); El Instituto Nacional de Ciencias Médicas y Nutrición Salvador Zubirán, México City, Mexico (INNSZ‐Mexico); and El Instituto de Medicina Tropical Alexander von Humboldt, Lima, Perú (IMTAvH‐Peru). CCASAnet sites that provided data only on cART‐treated patients were excluded.

Institutional review board approval was obtained locally for each participating site and for the CCASAnet data coordinating center (DCC) at Vanderbilt University, Nashville, TN, USA. In each of the sites contributing data to this study, except IMTAvH‐Peru, ethical regulations and policies permit retrospective analysis of de‐identified clinical data without informed consent when research is approved by an Institutional Review Board or appropriately constituted ethics committee. At IMTAvH‐Peru, patients consent at time of enrolment to provide de‐identified clinical data for research studies.

### 2.1 Study design and population

All cART‐naive adults (≥18 years) who were enrolled in participating CCASAnet sites during 2003 to 2017 and had a CD4 count of ≥350 cells/mm^3^ at enrolment were included in the study. Patients with history of an AIDS‐defining event or without information regarding AIDS‐defining events were excluded.

### 2.2 Primary outcome and statistical analysis

**Figure 1:**
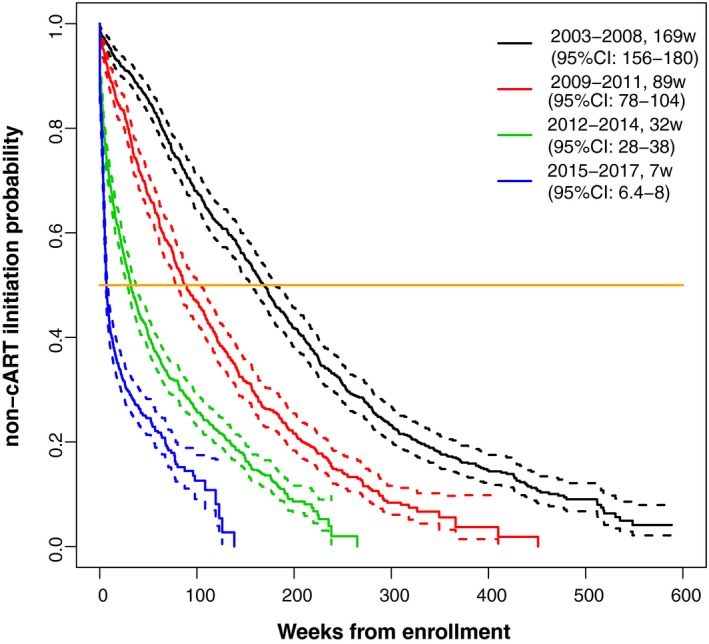
Kaplan‐Meier analysis of the probability of cART initiation over different periods of time among all patients.

The primary outcome of this study was cART initiation. Individuals were followed from cohort enrolment until cART initiation, death, lost to follow‐up (LTFU) defined as a >12‐month gap between their last medical visit date and closure date for each participant site, or cohort closure date. We estimated the unadjusted median time from enrolment to cART initiation by calendar year periods using Kaplan‐Meier methods among all patients and censoring at the end of their follow‐up time for those who did not initiate cART, were LTFU or died. Additionally, among patients who initiated cART, we used a median regression model to examine median times to cART initiation; 95% confidence intervals (CI) were computed using a nonparametric bootstrap with 10,000 replications. Using a Cox regression model, we calculated hazard ratios (aHR) and 95% CI for cART initiation by year of enrolment, adjusting by sex, age, CD4 count at enrolment, probable route of HIV transmission, and site of enrolment. Restricted cubic splines were used for continuous variables (year of enrolment, age, and CD4 count at enrolment) with three knots. The proportional hazards assumption was tested using Schoenfeld residuals; there was evidence that the hazards were not proportional, particularly with regards to the year of enrolment variable, although subsequent plots (e.g. Figure 1) suggested that this violation was not major. We used the results of the Cox regression to estimate and plot the adjusted probability of cART initiation within 30 days of enrolment by calendar year.

We also compared the sociodemographic (age at enrolment, sex and site of enrolment) and clinical characteristics (CD4 count at enrolment, year of enrolment, probable route of HIV transmission and years in follow‐up) of patients who never initiated cART (non‐cART initiators) with those who did (cART initiators), using the Wilcoxon rank sum and the chi‐square tests as appropriate. Additionally, we reported the last status available in the dataset for non‐cART initiators, classified as death, LTFU or administrative censoring at time of study closure.

All analyses were performed using RStudio Version 1.2.1335.

## 3 Results

### 3.1 Study population

We included a total of 3171 patients with CD4 ≥350 cells/mm^3^ and no AIDS defining event at enrolment. Of these, 2408 (75.9%) initiated cART during a median of 4.23 years of follow‐up. A description of the study population by clinical and sociodemographic characteristics is shown in Table 1. A total of 1650 patients (52%) had CD4 counts of >500 cells/mm^3^ at enrolment.

**Table 1:** Clinical and sociodemographic characteristics of patients enrolled in care in CCASAnet, 2003 to 2017

Characteristic	Combined (N = 3171)	cART initiators (N = 2408)	Non‐cART initiators (N = 763)	*p* value^a^
Age (years)	30 (25 to 37)	30 (25 to 38)	29 (24 to 36)	0.06
Male sex	2556 (80%)	1915 (80%)	641 (84%)	<0.007
CD4 at enrolment (cells/mm^3^)	508 (419 to 652)	498 (413 to 634)	542 (446 to 736)	<0.001
Viral load at enrolment (copies/mL)	23366 (5500 to 81958)	27392 (7578 to 92110)	11144 (1136 to 54292)	<0.001
Missing	73 (2.3%)	30 (1.2%)	43 (5.6%)	
Enrolled after 2013	1507 (47%)	1110 (46%)	397 (52%)	<0.001
Enrolled after 2015	840 (26%)	583 (24%)	257 (33%)	<0.001
Year of enrolment				
2003 to 2008	763 (24%)	173 (23%)	590 (24%)	<0.001
2009 to 2011	629 (20%)	133 (17%)	496 (20%)	
2012 to 2014	939 (29%)	200 (26%)	739 (31%)	
2015 to 2017	840 (26%)	257 (34%)	583 (24%)	
Probable route of HIV transmission				0.56
Heterosexual	1006 (31%)	776 (32%)	230 (30%)	
Men who have sex with men	1690 (53%)	1267 (52%)	423 (55%)	
Other^b^	387 (12%)	299 (12%)	88 (11%)	
Unknown	88 (3%)	66 (3%)	22 (3%)	
Clinic site				
Brazil	929 (29%)	780 (32%)	149 (19%)	<0.001
Chile	996 (31%)	663 (28%)	333 (43%)	
Honduras	50 (1%)	44 (2%)	6 (1%)	
Mexico	250 (7%)	220 (9%)	30 (3%)	
Peru	946 (29%)	701 (29%)	245 (32%)	
Time in follow‐up (years)	3.5 (1.3 to 6.5)	4.23 (2.01 to 7.3)	1.08 (0.25 to 3.11)	<0.001

Continuous variables are reported as medians (interquartile range). Percentages refer to their column.

a Wilcoxon rank sum test for continuous variables, Chi‐square test for categorical variables.

b Other route of HIV transmission includes eight injecting drug users (2.06% of the category).

Compared to non‐cART initiators, a higher proportion of cART initiators were female and a higher proportion had enrolled in recent years. The median CD4 at enrolment was lower among cART initiators than non‐cART initiators. Age and probable route of HIV transmission (*p* = 0.56) were fairly similar between those starting and not starting cART. Among those who never started cART, 33 (4.3%) were reported as deceased, compared to 54 (2.2%) patients who started cART (*p* = 0.003). Among those who died, the median times to death was 3.1 years and 3.9 years for those not starting and starting cART respectively (*p* = 0.09). Among those who never started cART, 461 patients (60%) were LTFU, whereas in the group that started cART, 211 (8.7%) were LTFU (*p* < 0.001). The median time in care prior to LTFU was 4.27 (interquartile range (IQR): 2.05 to 7.63) years for those who never started cART and 3.91 (IQR: 1.74 to 6.05) years for those who started cART. A total of 47 patients were known to have transferred to another clinic, 27 (1.1%) among the group who started cART and 20 (2.6%) among those who never started (*p* < 0.01).

The groups of deceased and LTFU people had a higher proportion of females: 28% and 26% respectively compared to 20% among those still in care. Median CD4 count at enrolment among LTFU was slightly lower (475, IQR: 393 to 584) compared to patients in care at end of follow‐up (501, IQR: 414 to 641), *p* = 0.002. Median CD4 count at last visit was also lower among LTFU (505, IQR: 364 to 672) compared to those in care at the end of follow‐up (671, 513 to 854), *p* < 0.01. Median CD4 count at enrolment among deceased patients was similar to that among surviving patients (471.5, IQR: 418.2 to 561.5 vs. 498, IQR: 413 to 635, *p* = 0.15), as was the median viral load at enrolment (23000, IQR: 7400 to 94000 vs. 27514, IQR: 7600 to 92066 respectively *p* = 0.56). The median time from ART to LTFU was 704 days (IQR: 206 to 1381) and patients who were not LTFU spent a median of 1120 days in care (IQR: 554 to 1894), *p* < 0.01.

### 3.2 Time to cART initiation

**Figure 2:**
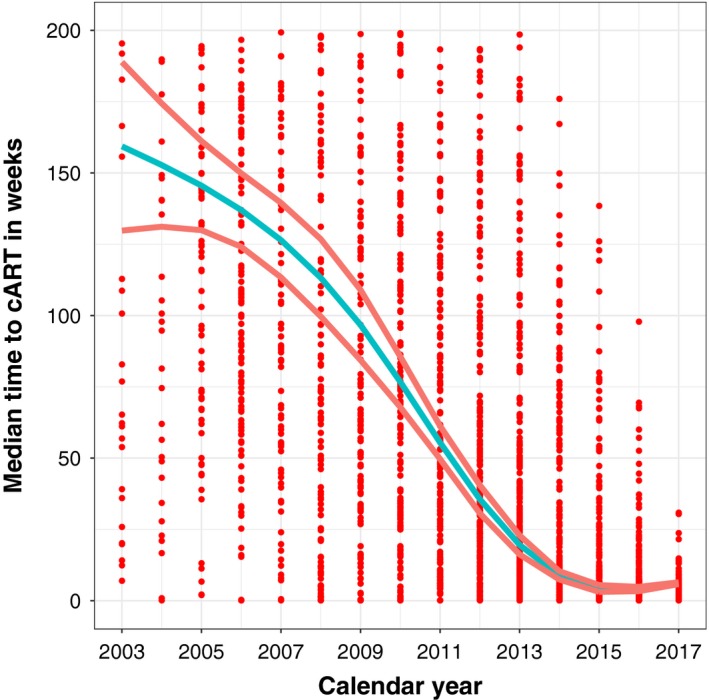
Median time to cART initiation over time for 2408 patients initiating cART, 2003 to 2016. The dots represent time to cART initiation for patients initiating cART based on the calendar year of enrolment. The curves represent the estimated median (95% confidence interval) based on median regression.

**Figure 3:**
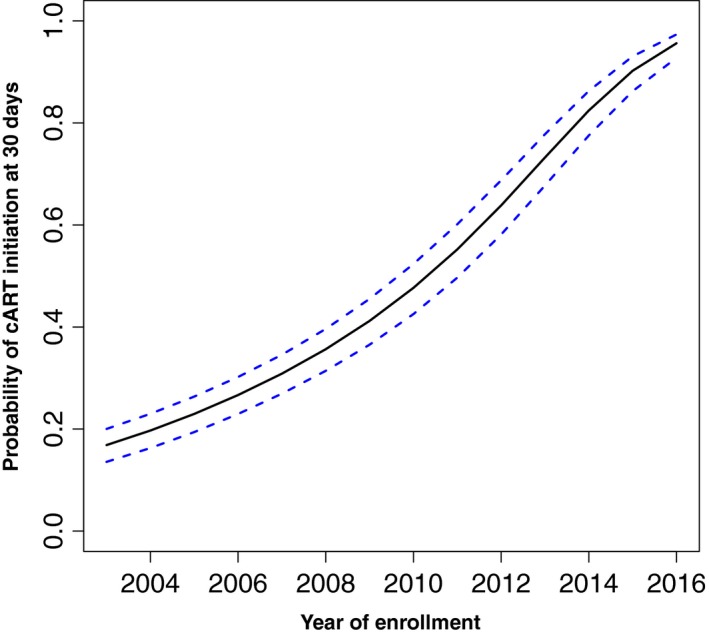
Adjusted probability of cART initiation within 30 days of enrolment by calendar year, 2003 to 2017.

Among all patients, median time to cART initiation between 2003 and 2008 was 169 weeks (95% CI: 156 to 180); time to cART initiation decreased to seven weeks (95% CI: 6.4 to 8) between 2015 and 2017 (Figure 1; Kaplan‐Meier estimates). Of those initiating cART, the unadjusted median time to cART initiation decreased from 156 weeks (95% CI: 65 to 225) in 2003 to 6.21 weeks (95% CI: 5.57 to 6.71) in 2013 and 4.71 weeks (95% CI: 4.28 to 5.14) in 2015 (Figure 2; median regression estimates). When compared to 2003, the adjusted instantaneous risk (i.e. hazard) of cART initiation was substantially greater in later years, in particular in 2013 (adjusted hazard ratio (aHR) = 7.14, 95% CI 5.84 to 8.73) and 2015 (aHR = 12.6, 95% CI 10.4 to 15.3); consequently, the probability of starting cART within the 30 days after enrolment in these patients also increased significantly over time (Figure 3; Cox regression estimates).

### 3.3 Other factors associated with earlier cART initiation

**Figure 4:**
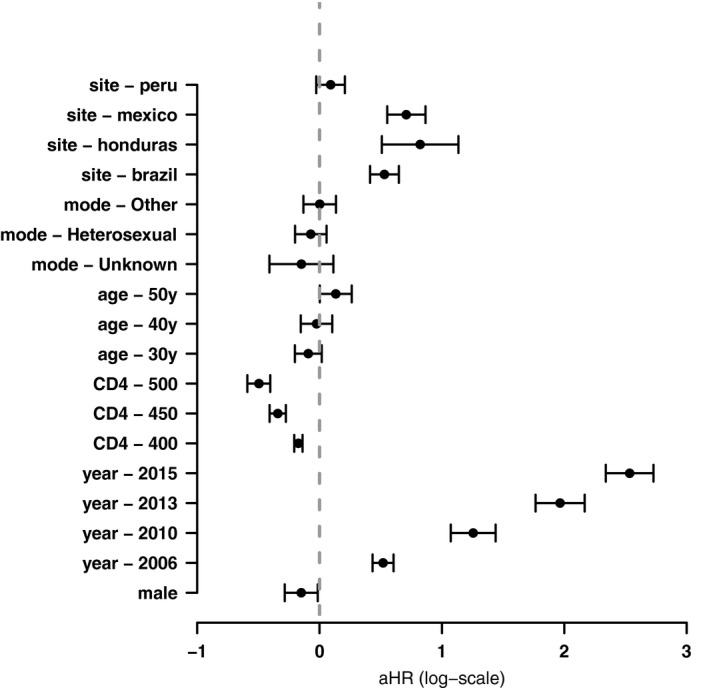
Adjusted hazard ratios of cART initiation, 2003 to 2017. Adjusted hazard ratios (aHR) presented on the log scale (i.e. as adjusted log hazard differences). The vertical dotted line is a log hazard difference = 0, which is equivalent to an aHR = 1 (a null association). The year 2003 is the reference enrolment year. A CD4 count of 350 cells/mm^3^ is the reference CD4 count category. Chile is the reference site for all other sites (Brazil, Honduras, Mexico, Peru). Men who have sex with men (MSM) and heterosexual refer to probable mode of HIV transmission. The reference age was 20 years.

Patients enrolled at our site in Brazil (aHR = 1.70, 95% CI: 1.51 to 1.91), Honduras (aHR = 2.27, 95% CI: 1.66 to 3.11) and Mexico (aHR = 2.03, 95% CI: 1.74 to 2.38) had an increased hazard of starting cART when compared to those enrolled in Chile. Male sex (aHR = 0.86, 95% CI: 0.75 to 0.99) and higher CD4 counts at enrolment (aHR = 0.61 comparing CD4 = 500 vs. CD4 = 350 cells/mm^3^, 95% CI: 0.55 to 0.67) were associated with a lower hazard of starting cART. After adjusting for other factors, age was not strongly associated with starting cART (*p* = 0.065) (Figure 4).

## 4 Discussion

In this study, we evaluated temporal trends and factors associated with cART initiation for patients enrolling in HIV care in a real‐world Latin American setting. The median time to cART initiation substantially shortened between 2003 and 2017, and the probability of initiating cART within 30 days of enrolment greatly increased. Later year of enrolment to care was the strongest predictor of cART initiation in our statistical models. These trends roughly reflect changes in guidelines over the years, which have progressively encouraged earlier cART initiation.

Other factors, such as high CD4 counts at enrolment, male sex and study site, were also associated with lower hazards of cART initiation. Among patients with CD4 counts >350 cells/mm^3^, higher CD4 counts at enrolment were associated with a lower hazard of cART initiation. This could suggest that clinicians may still delay cART initiation because of lower perceived risk of AIDS‐defining events in such patients, despite changes in guidelines. It may also be that difficulties in cART scale‐up across the Latin American region (for example, administrative barriers to implementing same‐day treatment) lead to treatment delay and prioritizing cART initiation in patients with more advanced disease. For example, Chilean clinical sites took longer to adopt newer WHO recommendations for cART initiation when compared with other countries of the region 19, 20 which supports the notion that implementation of guideline changes affected the decrease in time to cART initiation in our cohort.

Temporal trends in time to cART initiation in Latin America in asymptomatic patients with high CD4 cell counts have not been evaluated previously, though several studies have explored time to cART initiation overall, also showing trends towards earlier cART initiation over time 21, 22. Of note, most studies done looking at time to cART initiation look at CD4 count at the time of cART initiation, rather than median time (in days or years). Two clinical trials have shown that same day cART initiation has benefits in clinical outcomes such as engagement in care 11 and virological suppression 23. It is notable that 24% of our study population enrolling with high CD4 counts never started cART and that nearly two‐thirds of those were LTFU; the proportion LTFU was much higher among those who did not initiate cART than among those that did. Furthermore, in those who never started cART, 4% died, compared to 2% mortality in those who initiated cART, suggesting that asymptomatic patients not promptly initiating cART may be at higher risk of being lost and eventually dying. Therefore, same‐day cART may prove a crucial intervention in our region 24. In an additional analysis (not shown in the results) we found that more recent year of enrolment was associated with a lower hazard of the composite outcome of LTFU/death. However, this analysis is not able to distinguish between whether not starting cART led to LTFU/death or whether LTFU/death led to not starting cART (i.e. people who died or were LTFU did not have the same opportunities/follow‐up time to start cART). A careful study of this issue is beyond the scope of this manuscript.

Also in our study, it is notable that males were less likely than females to initiate cART. This finding has been shown elsewhere, including by our group in Latin America 13. In the Collaboration of Observational HIV Epidemiological Research Europe (COHERE) cohort, the probability of cART initiation was lower in migrant men, particularly in sub‐Saharan African migrants, compared to women 25. Caribbean male migrants to Europe were among the least likely to initiate cART. Similarly, in a cohort out of Tanzania, males started cART at a more advanced stage compared to females 26. In a systematic review evaluating sex disparities in HIV outcomes in the highly active antiretroviral therapy era from 1998 to 2013, there was a suggestion of improved survival of females compared to males in those starting cART 27.

In our study, the group of LTFU had a higher proportion of females, 26%, compared to 20% females among those still in care. However, in our study, as in a large study on gender and loss to follow‐up out of western Kenya (the AMPATH cohort) from 2001 to 2007, men with HIV infection were more likely to become lost to follow‐up than women, both before cART initiation and after. However, loss to follow‐up was more common before cART initiation than after in the AMPATH cohort 28. This was true in our study as well, suggesting that the time leading to cART initiation may be a crucial interval to retain people in care as well as to initiate a “return to health” for sick individuals. This was shown in South Africa, where initiating cART at a higher CD4 count resulted in a 26% to 42% reduced loss to follow‐up compared to those initiating at a lower CD4 cell count 22.

Our study had some additional important limitations. We did not include socioeconomic status, or other factors such as travel distance to the care facilities and administrative barriers at each medical site, which might account for some of the patients not initiating cART. Our database does not include information on causes of death. Our adjusted models also did not directly evaluate changes in times until cART initiation relative to the time of local implementation of the WHO guidelines, which makes effects of WHO guidelines within different sites more difficult to evaluate. However, the number of patients and the diversity of sites included make this study a source of useful information for the real‐world clinical care of PLWHA in Latin America. The temporal trends towards reducing the time to cART initiation, even in patients with high CD4 counts, indirectly supports the feasibility of earlier, or even same‐day, cART initiation, among PLWHA in Latin America.

## 5 Conclusions

Our study shows that the time to cART initiation has substantially decreased over calendar year in the Latin American region, roughly coinciding with evolving recommendations from the WHO to start cART earlier. However, a very high proportion of patients never started cART, compromising retention in care and survival, as shown by their higher proportion of LTFU and death. These data reinforce the notion that earlier treatment implementation strategies in the region could be needed.

## Competing interests

None.

## Authors’ contributions

BCR, YCV and AKP developed conceptualization and design of this study, contributed to interpretation of data, wrote the manuscript and had full access to all study data. YCV, PFR and BES conducted the data analysis, contributed to conception, design and interpretation of data and drafting the manuscript. YCV contributed to the data management. PFBZ, VV, CC, DP, EG, JSM and CM contributed to interpretation of data and revised the manuscript.
